# Structural, Physicochemical and Functional Properties of Protein Extracted from De-Oiled Field Muskmelon (*Cucumis melo* L. var. *agrestis* Naud.) Seed Cake

**DOI:** 10.3390/foods11121684

**Published:** 2022-06-08

**Authors:** Huijun Zhang, Runzhe Xu, Yushu Yuan, Xiuxiu Zhu, Wenhao Li, Xiangzhen Ge, Huishan Shen

**Affiliations:** 1 School of Life Science, Huaibei Normal University, Huaibei 235000, China; zhhuijun@126.com (H.Z.); xrz1577594325@163.com (R.X.); yuanyushu123@163.com (Y.Y.); z17856579357@163.com (X.Z.); 18393810565@163.com (X.G.); 2 College of Food Science and Engineering, Northwest A&F University, Xianyang 712100, China; liwenhao@nwsuaf.edu.cn

**Keywords:** field muskmelon seed protein, oil extraction methods, amino acid composition, physicochemical properties, functionality

## Abstract

For oil plants, the oil extraction method is a crucial factor in influencing the functional characteristics of the protein. However, reports of protein functionality as affected by the oil extraction process are scarce. In this study, field muskmelon seed (FMS) protein was extracted by Soxhlet extraction method (SE), organic solvent extraction method (OSE), aqueous extraction method (AE), and pressing extraction method (PE), and its structure, amino acid profile, physicochemical properties, and functionality were determined. Molecular weight distribution was similar for all FMS proteins, whereas protein aggregates contents were most excellent for SE and OSE. FMS protein comprised predominantly glutamic acid, leucine, aspartic acid, arginine, and proline. Total amino acids content was highest for SE. Differences in functionality between four FMS proteins for different oil extraction methods were vast. PE had the highest value of solubility, and AE exhibited the lowest. AE had the greatest water and oil holding capacity. PE presented better foaming and emulsion capacities than other samples. This study demonstrated that the extraction oil method could impact the protein’s physicochemical and associated functional characteristics. High-quality plant oil and protein could be simultaneously obtained by modulating the oil extraction method in future research.

## 1. Introduction

The increasing world population annually consumes large amounts of melon fruits. While consuming and processing of melon fruits, a great deal of by-products, such as peels and seeds, are often considered waste to discard [1]. However, the seeds possess abundant valuable nutrients and bioactive components [2]. Indeed, utilizing food by-products to develop high-quality products effectively reduces food loss and waste. Thus, the academy has a growing interest in extracting and purifying desired compounds from melon fruits by-products.

Field muskmelon (*Cucumis melo* L. var. *agrestis* Naud.) is an annual herbaceous plant belonging to the Cucurbitaceae family [3]. It is a wild melon with a great edible value generally distributed and cultivated in Africa, China, and India. It also exhibits excellent medicinal properties, such as antioxidant, anti-inflammatory, analgesic, and hypoglycemic effects [4]. Field muskmelon seeds (FMS) are yellowish, long elliptic with smooth surfaces. The seeds are a promising oil crop that can be used to produce edible oils because they contain large plenty of fats. The oil extracted from FMS has high contents of γ-tocopherol, α-tocopherol, β-sitosterol, and phenolic compounds [5]. Indeed, many FMS cakes are generated as a by-product during oil production and unutilized. Nevertheless, the de-oiled cakes still contain many nutrients and proteins. The de-oiled FMS cakes can be used as a protein source for human consumption, making full use of this by-product and creating more economic values.

Protein is an essential food ingredient with valuable nutrition, specific physicochemical properties, and essential functionality [6]. Protein obtained from plants can be used in processed foods in place of meat proteins and can increase the nutritional quality and meliorate the sensory properties such as texture, flavor, and color of protein-rich foods [7,8].

The composition and functional properties of protein are influenced by the extraction method, extraction conditions (pH, temperature, solid-liquid ratio, mixing time), isolation techniques (ultrafiltration, dialysis, precipitation), and drying method (freeze, spray, and vacuum drying) [9,10,11,12,13]. For oil crops, the oil extraction process significantly affects the structure and functionality of protein [14]. Liu et al. [15] reported that oil extraction processing influenced the component, molecular weight distribution, solubility, foaming stability, emulsifying stability index of peanut protein. Under different oil extraction methods, the deformation of the cell walls and the destruction of the cytoplasmic networks are inconsistent. These changes may denature the proteins and differ the protein functional properties. Previous studies focused on improving the oil extraction technologies to enhance the yield and quality of oil, but studies on the by-product of oil extraction are scarce.

In our previous study, we extracted the oil of FMS through the Soxhlet extraction (SE), organic extraction (OSE), aqueous extraction (AE), and press extraction (PE) method [16]. It was demonstrated that the SE showed the highest oil yield (34.47%), the strongest ferric reducing antioxidant power and the free radical scavenging ability of DPPH and ABTS+. Therefore, this work is intended to be a continuation of the aforementioned study. In this study, FMS protein was obtained from de-oiled FMS cakes, and systematically investigate the impacts of conventional oil extraction processing on the structural, amino acid composition, physicochemical and functional properties of protein. The results could increase the added value of the by-products of melon fruit seeds.

## 2. Materials and Methods

### 2.1. Materials

FMS was obtained from Huaibei Normal University (Anhui, China). Sodium dodecyl sulfate (SDS), glycine, ethylene diamine tetraacetic acid (EDTA), 5,5´-Dithio-bis 2-nitrobenzoic acid (DTNB), protein marker (14.4–97.4kD) and standard protein (66–669 kDa) were purchased from Beijing Solarbio Science and Technology Co., Ltd., China. The water used in this study is ultrapure water. All other chemicals were of analytical grade.

### 2.2. Oil Extraction Method

#### 2.2.1. Soxhlet Extraction Method (SE)

FMS oil was extracted with SE as described by Özcan et al. [17]. Briefly, FMS was ground and put into a paper sleeve, then extracted using petroleum ether in a Soxhlet extractor at 40 °C for 6 h. After desolventizing, the de-oiled cakes were collected for protein extraction.

#### 2.2.2. Organic Solvent Extraction Method (OSE)

The OSE of oil was produced using a modified method of Liu et al. [15]. The ground FMS was mixed with n-hexane at a ratio of 1:10 with constant stirring in a magnetic stirrer (55 °C) for 3 h, then centrifugated at 4000× *g* for 20 min. The n-hexane was removed from the precipitated cakes by a rotary vacuum evaporator at 50 °C. The de-oiled cakes were collected for protein extraction.

#### 2.2.3. Aqueous Extraction Method (AE)

The AE was followed by Khoei and Chekin [18]. First, the ground FMS and water with the ratio of 1:10 were mixed, and the solution was stirred for 3 h at 50 °C. Then the solution was centrifuged at 5000× *g* for 20 min. Next, the collected oil and cream were stored at −20 °C for 24 h. After thawing, the samples were centrifuged at 4000× *g* for 10 min to break emulsion until oil appeared. Finally, the dried precipitated de-oiled cakes were collected for protein extraction.

#### 2.2.4. Pressing Extraction Method (PE)

The FMS was loaded into the DH-50 screw oil press that had been preheating. Then, the extracted oil was centrifuged at 3000× g for 10 min to remove solid impurities and stored at 4 °C. Finally, the de-oiled cakes were collected for protein extraction.

### 2.3. Preparation of FMS Protein

Proteins were isolated from de-oiled FMS cakes using the method of Malik et al. [19] with slight modification. De-oiled cakes were mixed with water at a ratio of 1:10, the pH of the mixture was adjusted to 10 with 1 M NaOH and stirred for 1 h at 50 °C. Then the mixture was centrifuged at 6000× *g* for 15 min, the supernatant was collected, and sediment was extracted again. The obtained supernatant was adjusted to pH 4.5 using 1 M HCl and stood for 30 min. Finally, centrifugated sediments were collected, washed with water to neutral and freeze-dried.

### 2.4. Determination of FMS Protein Yield and Proximate Composition

The yield of protein sample was calculated as described by Mir et al. [11]. The calculation formula was as follows:

(1) $$Yield\;\left(\%\right)=\frac{weight\;of\;protein\;\left(g\right)}{weight\;of\;FMS\;seeds\;\left(g\right)}\times 100$$

The contents of protein, moisture, fat were determined by the standard methods of AOAC (2006).

### 2.5. Amino Acid Composition Analysis

The amino acid composition was performed as Kaushik et al. [20] described. The protein samples were hydrolyzed with 6 M HCl in a hydrolysis tube (three drops of phenol were added) for 22 h at 110 °C. After cooling and filtration, 1 mL of the filtrate was dried under reduced pressure. About 1 mL sodium citrate buffer (pH 2.2) was mixed with the samples, and then the mixture was filtered through a 0.22 µm filter. The samples were determined by an amino acid analyzer (Biochrom 30+, Britain).

### 2.6. Structural Characterization

#### 2.6.1. Scanning Electron Microscopy (SEM)

A speck of protein sample was fixed to a slab and sprayed with a coat of metal. All samples were recorded with a Nova Nano SEM 450 SEM (FEI, USA).

#### 2.6.2. Sodium Dodecyl Sulfate–Polyacrylamide Gel Electrophoresis (SDS-PAGE)

SDS-PAGE analysis of protein was performed using 5% stacking gels and 12% separating gels as described by Malik et al. [19]. Protein sample (10 mg) was dissolved in 1 mL of sample buffer (0.0625 M Tris-HCl, 10% glycerol, 2% SDS, and 5% β-mercaptoethanol, 0.0025% bromophenol blue). The sample was heated at 100 °C for 5 min and then centrifuged at 8000× *g* for 10 min. Sample (10 μL) was loaded on a DYY-12 vertical slab of gel with a thickness of 1.5 mm (Liuyi Biological Technology Co., Ltd., Beijing, China). The stacking gel and separating gel were run at 80 V and 110 V, respectively. After staining and decolorizing, the gel was observed with Image Lab software.

#### 2.6.3. Molecular Weight (Mw) Distribution

The Mw was determined by high-performance liquid chromatography (HPLC, LC-20A, Shimadzu, Japan) adopting the method of Cui et al. [21]. The protein was dispersed to 10 mg/mL with the phosphate buffer (10 mM, pH 7.0) and filtered for HPLC analysis through a 0.45 μm filter. Shodex Protein KW-804 column (8.0× 300 mm, Showa, Kyoto, Japan) was selected for the test. The HPLC conditions were: injection volume, 50 μL; mobile phase, 10 mM phosphate buffer (pH 7.0) containing 0.3 M NaCl; flow rate, 1 mL/min. The eluate was measured at 280 nm in a diode array detector. Standard protein (66–669 kDa) was used for calibration. The standard curve was established as a linear relationship between the retention time (t) and the logarithm of the Mw: logMw=−0.1506t + 6.7156, R^2^ = 0.9920. The relative amount of protein aggregates and non-aggregates was calculated as follows:

(2) $$C_{\mathrm{aggregates}}(\mathrm{mg}/\mathrm{mL})=\frac{{\mathrm{PA}}_{\mathrm{aggregates}}}{\mathrm{TA}}{\;\times \;C}_{\mathrm{solution}}$$

(3) $$C_{\mathrm{non-aggregates}}{(\mathrm{mg}/\mathrm{mL})=C}_{\mathrm{solution}}-{\;C}_{\mathrm{aggregates}}$$

where PA_aggregates_ is the peak areas of protein aggregates, TA is the total areas of protein, C_solution_ is the concentration of protein sample.

#### 2.6.4. Free Sulfhydryl Group (SH) contents

The SH content was determined as described by Zhao et al. [22]. Protein samples (100 mg) were solubilized with 10 mL of Tris-Gly buffer (0.086 M Tris, 0.09 M glycine, 0.004 M EDTA, pH 8.0) and stirred for 30 min on a magnetic stirrer. After centrifuged at 8000× g for 10 min, 4 mL supernatant was solubilized with 160 μL Ellman’s reagent (4 mg/mL, DTNB in Tris–glycine buffer) and the absorbance was measured at 412 nm after 5 min using an ultraviolet-visible spectrophotometer (Shanghai Youke Instrument Co., Ltd., China). The reagent buffer was used as the blank. The calculation formula was as follows:

(4) $$\mathrm{SH}\;(\mu \mathrm{mol}/g)=\frac{73.53\;\times \;A\;\times \;D}{C}$$

where, A is the absorbance at 412 nm, D is the dilution factor, C is the protein concentration (mg/mL).

#### 2.6.5. Protein Intrinsic Fluorescence

The fluorescence spectrum was determined as described by Feng et al. [23]. The protein solution (1 mg/mL) was prepared with 10 mM phosphate buffer (pH 7.0) and recorded using a fluorescence spectrophotometer (LS55, PerkinElmer, Waltham, MA, USA) at room temperature. The excitation wavelength was 280 nm, and the emission spectra were recorded from 290 to 500 nm. A slit of 5 nm was set for emission.

### 2.7. Differential Scanning Calorimetry (DSC)

About 4 mg of protein sample was placed into an aluminum pan and measured by a Q2000 DSC (TA Instruments, New Castle, DE, USA). It operates in temperatures from 30 °C to 200 °C. The heating rate was 10 °C/min.

### 2.8. Functional Properties

#### 2.8.1. Solubility

Protein solubility was determined by the method of Stone et al. [24]. Protein solution (1%, *w*/*v*, pH = 7) was stirred on a magnetic stirrer at room temperature for 1 h and then centrifuged at 4000× *g* for 15 min. The protein content of the supernatant was measured using a Coomassie brilliant blue method [25].

(5) $$\mathrm{Solubility}\;(\%)=\frac{\mathrm{protein}\;\mathrm{content}\;\mathrm{in}\;\mathrm{the}\;\mathrm{supernatant}}{\mathrm{total}\;\mathrm{protein}\;\mathrm{content}}\;\times \;100$$

#### 2.8.2. Water and Oil Holding Capacity

The water holding capacity (WHC) and oil holding capacity (OHC) were determined as described by Ghribi et al. [26]. Approximately 0.4 g protein sample was dispersed in 4 mL water (or soybean oil) and placed in a 10 mL centrifuge tube. The dispersions were vortexed for 1 min and kept for 30 min at room temperature, followed by centrifugation for 20 min at 3000× *g*. Then, the supernatant was removed and weighed the increase in weight of the protein. The calculation formula was as follows:

(6) $$\mathrm{WHC}\;\mathrm{or}\;\mathrm{OHC}\;(g/g)=\frac{W_{2}-{\;W}_{1}}{W_{0}}$$

where, W_2_ is the weight (g) of the tube with the protein and absorbed water (soybean oil), W_1_ is the weight (g) of the tube and protein. W_0_ is the weight (g) of the protein.

#### 2.8.3. Foaming Capacity and Stability

The foaming capacity (FC) and foaming stability (FS) were determined as described by Das et al. [27]. In Brief, 0.5 g of protein sample was dissolved in 20 mL phosphate buffer (0.01 M, pH 7) and then homogenized by an XHF-D high-speed disperser (Ningbo Scientz Biotechnology Co. Ltd, Zhejiang, China) for 1 min. The blend was immediately transferred into a graduated cylinder and recorded the foam volume (V_0_). Then the foam volume was recorded after 30 min (V_1_). The calculation formula was as follows:

(7) $$\mathrm{FC}\;(\%)=\frac{V_{0}}{20}\;\times \;100$$

(8) $$\mathrm{FS}\;(\%)=\frac{V_{1}}{V_{0}}\;\times \;100$$

#### 2.8.4. Emulsion Activity and Stability

The emulsion activity index (EAI) and emulsion stability index (ESI) were determined as described by Ghribi et al. [26]. In Brief, 0.1 g of protein sample was dissolved in 10 mL phosphate buffer (0.01 M, pH 7), then mixed with 10 mL soybean oil and homogenized by XHF-D high-speed disperser (Ningbo Scientz Biotechnology Co. Ltd, Zhejiang, China) for 1 min to produce the emulsion. The 100 μL emulsion was pipetted from the bottom of the container at 0 and 10 min after homogenizing and mixed with 5 mL of 0.1% SDS. The absorbance of emulsions was measured at 500 nm with an ultraviolet-visible spectrophotometer (Shanghai Youke Instrument Co., Ltd., Shanghai, China). The calculation formula was as follows:

(9) $${\mathrm{EAI}\;(m}^{2}/g)=\frac{2\times 2{.303\;\times \;A}_{0\;}{\times \;D}_{F}}{c\times \varphi \times 10000}$$

(10) $$\mathrm{ESI}\;(\min)=\frac{A_{0}}{A_{0}-{\;A}_{10}}\;\times \;\Delta t$$

where, A_0_ is the absorbance of the diluted emulsion immediately after homogenization, A_10_ is the absorbance of the diluted emulsion after homogenization 10 min, D_F_ is the dilution factor, c is the sample concentration (g/mL), φ is the oil volume fraction of the emulsion, Δt is 10 min.

### 2.9. Statistical Analysis

All the data were expressed as mean ± standard deviation. Analysis of variance (ANOVA) was performed by Duncan’s test (*p* < 0.05) using SPSS 21.0 (SPSS Inc., Chicago, IL, USA).

## 3. Results and Discussion

### 3.1. Yield and Proximate Composition of FMS Protein

The yield and proximate composition of FMS protein concentrate are presented in Table 1. The oil extraction method showed a notable effect on the yield of FMS protein, with SE possessing the highest protein yield (16.7%) and PE showing the lowest yield (3.3%). In case of PE, mechanical pressing the FMS breaks down their cell walls and force oil to be squeezed out of plant cells. The high residual oil rate of the FMS cakes caused by insufficient extrusion in PE process may be responsible for the low yield.

SE presented the highest protein (87.49%) and moisture content (11.03%), while the lowest fat value (0.62%). AE had minimum protein (71.00%) and moisture content (5.04%), and high amounts of fat (13.59%). During the AE process, oil-rich emulsion fraction, protein-rich liquid fraction, and insoluble fiber-rich solid fraction appeared after centrifugation. The residual oil in precipitated cakes was determined mainly by separation efficiency. The residual oil in the protein-rich liquid fraction caused the stabilization of insoluble particles [15]. Thus, AE possessed lower a protein quantity and higher fat content than other oil extraction methods.

### 3.2. Amino Acid Composition Analysis

The amino acid profiles of FMS protein extracted by different oil extraction methods are presented in Table 2. Significant variations in amino acid composition were found among the four FMS proteins. SE exhibited the highest total amino acids (803.28 mg/g), followed by OSE (711.67 mg/g) and PE (656.88 mg/g), while AE had the lowest (650.67 mg/g). Total essential amino acid contents of PE (293.18 mg/g) were lower than the other three FMS protein samples, which might be attributed to Strecker degradation and the Maillard reaction consumed amino acids during the pressing extraction process [28].

Glutamic acid was the most abundant in four FMS proteins, and the content was 125.61 mg/g for SE, 111.17 mg/g for OSE, 104.51 mg/g for AE, and 119.56 mg/g for PE; while cysteine was low in both four FMS proteins (2.83–4.16 mg/g). Additionally, four FMS proteins were rich in leucine, aspartic acid, arginine, and proline, and the total amount of these four amino acids was 381.75 mg/g for SE, 337.14 mg/g for OSE, 313.03 mg/g for AE, and 329.05 mg/g for PE.

The cholesterolemic and atherogenic effects of a protein can be measured by the ratio of lysine to arginine. A protein with a lesser lysine-arginine ratio exhibits lower lipidemic and atherogenic effects [20]. FMS proteins presented a lysine-arginine ratio of 0.44 (SE), 0.43 (OSE), 0.41 (AE), and 0.36 (PE), respectively. Compared with chickpea protein (0.82), soy protein (0.80), and whey protein (5.45) [26,29], the lesser lysine-arginine ratio suggests that FMS proteins might be a valuable protein for cardiovascular health.

### 3.3. Structural Analysis

#### 3.3.1. Surface Morphology

**Figure 1:**
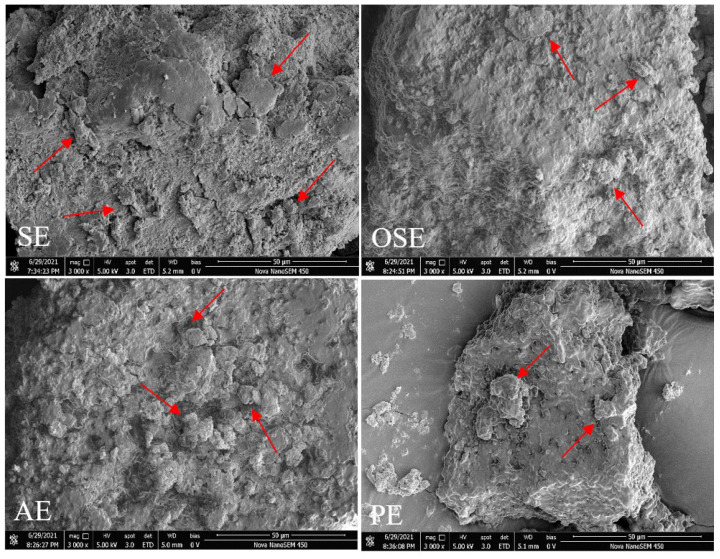
SEM images of field muskmelon seeds protein extracted by different oil extraction methods.

The SEM images of the FMS protein are displayed in Figure 1. During the freeze-drying process, protein-protein interactions including hydrophobic, electrostatic and covalent linkages enhanced and resulted in solute aggregation. Therefore, the surface of all proteins was a continuous space structure and presented a sheet-like structure. Moreover, some roughened clusters (red arrows) can be observed on the protein’s surface. There was no visible difference among SE, OSE and AE samples. However, PE sample showed an irregular shape and rough surface, which indicated that thermomechanical treatment significantly destroyed the protein structure. Liu et al. [15] reported that the peanut protein after different oil extraction processing showed consistent surface morphology, while drying methods affect significantly the surface morphology.

#### 3.3.2. SDS-PAGE

**Figure 2:**
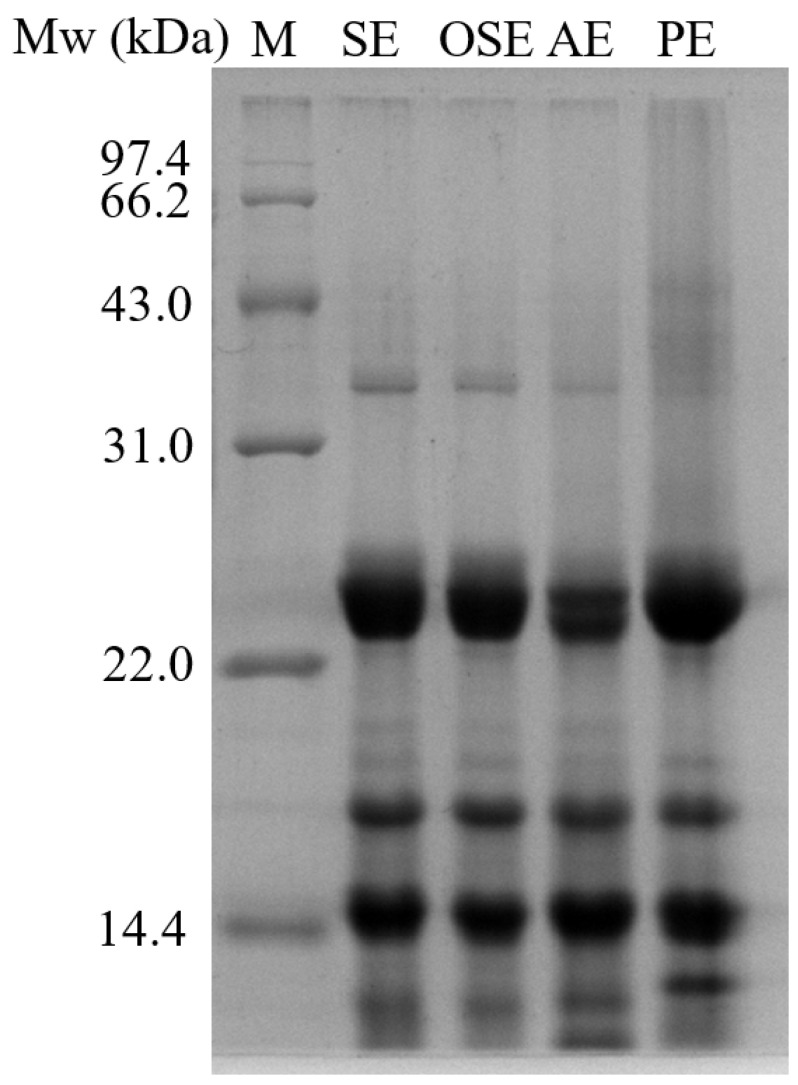
SDS-PAGE of field muskmelon seeds protein extracted by different oil extraction methods.

The SDS-PAGE profile of FMS protein is shown in Figure 2. The electrophoretic profiles of SE and OSE showed large similarities, which contained major bands with 36, 23–26, 18–20, and 7 kDa. Compared with SE and OSE, AE had very low concentration of low Mw subunits (36, 20, and 19 kDa), but a new band appeared at the bottom. The three prominent bands with 36, 20, and 7 kDa of PE disappeared compared with other protein samples, whereas bands with 9 kDa appeared. This phenomenon may be due to high-temperature pressing process altering the aggregation state of the protein. The electrophoresis profile suggested that FMS protein mainly contains low Mw polypeptide subunits.

#### 3.3.3. Molecular Weight (Mw) of FMS Protein

**Figure 3:**
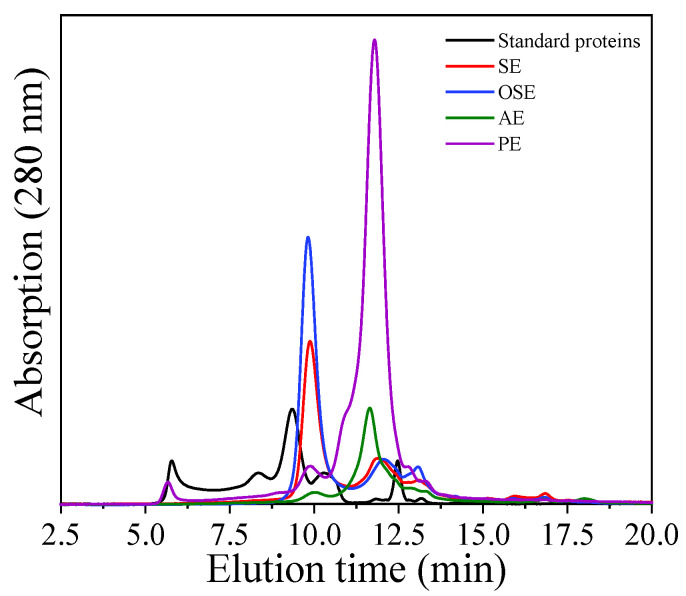
Molecular weight distribution of field muskmelon seeds protein extracted by different oil extraction methods.

The Mw profile of FMS protein measured by HPLC is shown in Figure 3. The elution profile of four proteins showed two major peaks, corresponding to aggregates and non-aggregated protein, respectively. As shown in Table 1, the Mw of SE, OSE, AE and PE was 169.33 kDa and 84.54 kDa, 172.89 kDa and 79.95 kDa, 162.03 kDa and 91.66 kDa, 168.92 kDa and 87.31 kDa, respectively. There is no notable difference in Mw distribution between four proteins, suggesting that different oil extraction methods hardly affect FMS protein’s Mw distribution.

However, the process of oil extraction significantly impacted the contents of FMS protein. SE and OSE contain high levels of protein aggregates (8.01 and 7.29 mg/mL) and small amounts of protein non-aggregates (0.61 and 0.43 mg/mL), whereas AE and PE showed opposite results. It indicated that the organic reagent (petroleum ether and n-hexane) could cause the peak of protein non-aggregates to shift to higher Mw.

#### 3.3.4. Free Sulfhydryl Group (SH) Contents

The sulfhydryl group participates in weak secondary bonds (i.e., disulfide bonds) and plays a vital role in stabilizing protein conformation and maintaining protein activity. Variations in SH group contents can reflect the degree of protein denaturation [30]. From Table 3, there are significant differences in free SH group contents among four proteins. The highest free SH content was observed in PE (12.6 μmol/g), followed by AE (8.86 μmol/g) and OSE (4.78 μmol/g), and SE had the lowest SH content (3.42 μmol/g). Furthermore, the SH group might participate in the formation of aggregates, which was confirmed in the Mw of protein (Table 1). The higher content of the free SH group in PE may be because high temperature destroyed the disulfide bonds and converted them to free SH. On the other hand, high temperature promotes the unfolding of the protein and internal SH group, which originally existed in the hydrophobic structure of the protein molecule exposed [9].

#### 3.3.5. Protein Intrinsic Fluorescence

The aromatic amino acids of protein, such as phenylalanine (Phe), tyrosine (Tyr) and tryptophan (Trp), are solvatochromic. Therefore, fluorescence intensity and maximum emission wavelength (λ_max_) are related to the polarity of the microenvironment and change with the unfolding of protein and exposure of the chromophores. Therefore, the intrinsic fluorescence spectrum can monitor the tertiary structure change of protein [15,31].

**Figure 4:**
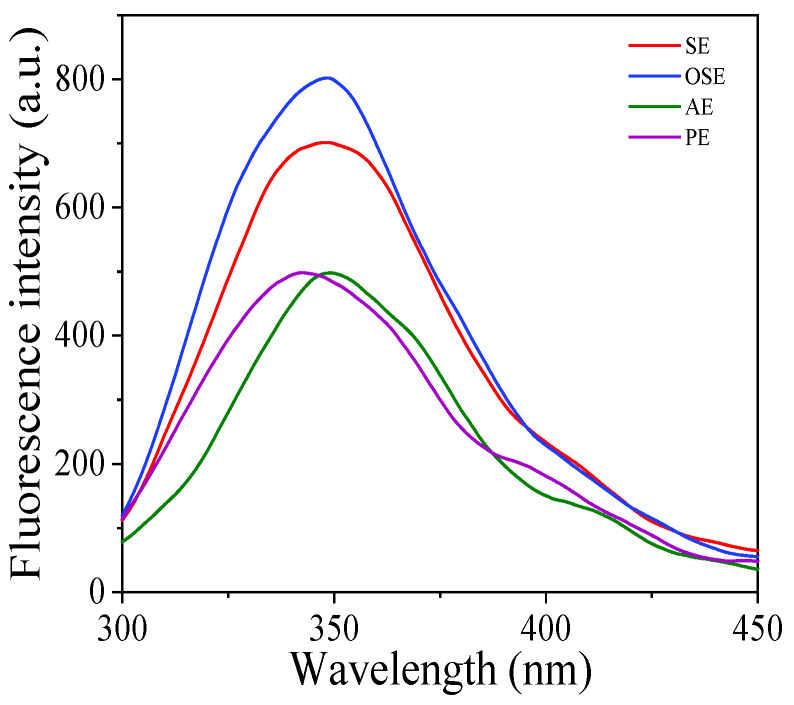
Intrinsic fluorescent spectrum of field muskmelon seeds protein extracted by different oil extraction methods.

From Figure 4, the λ_max_ of SE, OSE and AE was closed to 349 nm, whereas the λ_max_ of PE had a blue shift to 344 nm, showing that the aromatic amino acids in PE were transferred into a hydrophobic environment [32,33]. It was in line with the result of amino acids, the tyrosine and phenylalanine contents in PE were lowest (Table 2). This phenomenon revealed that the oil extraction method could alter the tertiary structures and aggregation state of FMS protein. From Figure 4, the fluorescence intensity of SE and OSE was greater than that of AE and PE, suggesting organic solvent extraction (petroleum ether and n-hexane) contributed to the exposure of aromatic amino acids and created a more compact structure of the protein. On the other hand, the low fluorescence intensity of PE may be due to the unfolding of protein during the pressing process [31]. The previous study of Liu et al. [15] reported that the fluorescence intensity of peanut protein obtained by solvent extraction was higher than that of aqueous extraction.

### 3.4. Differential Scanning Calorimetry (DSC)

**Figure 5:**
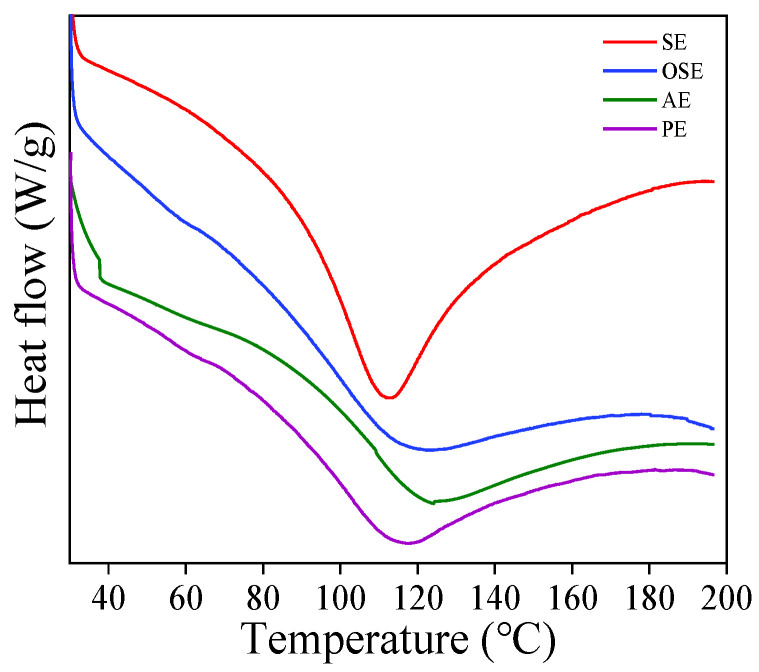
DSC thermogram of field muskmelon seeds protein extracted by different oil extraction methods.

The thermal properties of four FMS protein samples were displayed in Figure 5, and the onset (T_o_), denaturation (T_d_), endset temperature (T_e_) and enthalpy (ΔH) are shown in Table 3. T_d_ refers to the denaturation temperature of protein and indicates thermal stability. ΔH is the energy required to induce the denaturation of protein molecules [27].

As seen from Figure 5, a prominent endothermic peak was observed for the protein samples with denaturation temperature ranging from 116 to 124 °C. The highest T_d_ was found for AE (124.16 °C), followed by SE (118.22 °C) and OSE (119.03 °C). PE was less thermally stable, with a T_d_ of 116.13 °C. Variation in the thermal stability may be attributed to the changes in protein structure and conformation, amino acids composition, protein-protein interactions during different oil extraction processes [28]. The lower thermal stability of PE suggests greater structural and conformational changes during pressing treatment.

The ΔH of SE, OSE, AE, and PE varied from 82.94 to 184.85 J/g, and AE had the lowest ΔH, suggesting that it needs less energy for denaturation. In addition, the phenomenon indicated that the freeze treatment (−20 °C) during the aqueous extraction process disrupted the intermolecular bonds, resulting in the AE sample’s compact structure being lost and increased protein unfolding.

### 3.5. Functional Properties

#### 3.5.1. Solubility

Solubility is one of the most critical functional properties of the protein, because it affects protein’s other functionality and influences the color, texture, and sensory properties of the protein products [27,32]. In addition, solubility results from the equilibrium between protein-protein and protein-water interactions [8].

As shown in Table 4, different oil extraction methods produced FMS proteins that presented different degrees of solubility at pH 7.0, and PE exhibited the highest solubility (17.87%). The great solubility might be due to the sizeable insoluble protein aggregates dissociated into soluble non-aggregates during the pressing extraction, as illustrated in Table 1. Moreover, the looser tertiary structure is responsible for the high solubility by promoting the intramolecular hydration of proteins [22]. On the other hand, AE had the lowest solubility of 10.65%, resulting from incomplete centrifugation of protein fractions. During the AE process, the protein was transferred from the liposome into the water phase after centrifugation, and limited separation efficiency kept soluble protein in the aqueous solvent [15]. On the other hand, the residual oil could reduce the protein-water interaction, which decreased the solubility of FMS protein. The lower solubility of SE may be due to the presence of more hydrophobic groups, which becomes exposed and ultimately results in lower solubility values.

#### 3.5.2. Water and Oil Holding Capacity

WHC and OHC refer to the number of water and oil absorbed by the protein. They can improve the flavor, enhance the texture and influence the quality characteristics of food products [6]. According to Table 4, the WHC and OHC significantly differed among four proteins, AE showed the highest WHC (1.48 g/g), followed by SE (1.31 g/g) and OSE (1.24 g/g), PE had the lowest WHC (0.91 g/g). The aqueous extraction method caused more hydrophobic sites buried in the internal structure of the protein; thus, AE had the highest WHC. The result was consistent with the reports of Liu et al. [15], in which they have shown that aqueous extraction processing enhanced the WHC of peanut protein than other oil extraction methods. Protein with plentiful hydrophilic groups on the surface can absorb more water [12]. The low WHC of PE was attributed to the aromatic amino acid residues were exposed in a hydrophobic environment, which was in line with the result of intrinsic protein fluorescence (Table 3). The WHC of FMS protein was comparable to the reported values for peanut protein (0.37–2.93 g/g) and hemp protein (0.80–1.59 g/g) [15,34]. However, FMS protein displayed lower WHC values compared to red lentil protein and pea protein [6,12], indicating the presence of less hydrophilic groups present in its structure than in the other proteins.

From Table 4, AE displayed greater OHC (1.36 g/g) than other protein samples (0.97–1.07 g/g). The changes of the hydrophobic groups in proteins can increase their OHC. The protein hydrophobic groups-lipid interactions that exist in AE enhance its OHC. Comparable values for OHC were observed in pea protein (1.07–1.40 g/g) [24], whereas the OHC values measured in our study were lower than the peanut protein (2.93 g/g) obtained by a similar aqueous extraction method [15]. Differences in OHC may be because of the different protein separation methods, protein structure, and size, etc.

#### 3.5.3. Foaming Capacity and Stability

FC refers to the ability of protein solution to produce foams under certain conditions. FS represents the capacity of the protein to maintain the foam over a defined period [12]. Foaming properties are crucial for foods that needed higher whipping and aeration characteristics. FC mainly depends on the protein solubility, interface diffusion speed, interfacial tension, and other components (carbohydrates, moisture and fat). FS is generally influenced by intermolecular interactions and the cohesiveness of protein molecules [26].

The FC and FS of protein obtained from different oil extraction processing are shown in Table 4. The FC and FS were in the range of 12.50–21.67% and 17.22–64.64%, respectively, markedly different (*p* < 0.05). SE and PE presented higher FC and FS, whereas AE had the lowest FC and FS (12.50% and 17.22%). The higher FC and FS in PE may be because the unfolding of protein in the pressing process caused more hydrophobic regions to be exposed [28]. Moreover, the partial denaturation of the protein increased the rigidity of the interfacial film, thus enhancing the foam stabilization during pressing process.

On the other hand, SE showed more excellent FC and FS, possibly attributed to its higher λ_max_ and lower fat content. Liu et al. [15] reported that the presence of fat in peanut protein could reduce the surface tension of the foam and hydration capacity, resulting in lower FC and FS. Therefore, reducing the residual oil content in cakes would increase the foaming properties of the protein.

We compared the foaming properties of FMS protein with other proteins due to limited research on the FMS protein. One of the important conditions for achieving good FC and FS is a higher protein solubility, because a higher solubility increases the viscosity of the solution, making the foam structure stiffer and more stable. The FC and FS of FMS protein in this study were found to be lower than red lentil protein, kidney bean protein, field pea protein, and pea protein [6,7,9], because the solubility of FMS protein was lower than soy protein. Variation in the FC and FS of different protein are associated with protein type, extraction procedure, pH, and drying method. Das et al. [27] reported that the foaming properties of protein presented greatly pH dependence. Proteins exhibited the lowest FC at the pH close to the isoelectric point, because the protein was less soluble at its isoelectric point. Soluble proteins can adsorb on the air-water interface and decrease the surface tension by unfolding and interacting with other protein molecules, causing improved foaming characteristics [35].

#### 3.5.4. Emulsion Activity and Stability

The emulsifying properties of protein include EAI, which is the ability of the protein to form an emulsion, and ESI, which indicates the stability of the protein emulsion over time [36]. EAI is associated with the protein’s ability to absorb on the oil-water interface, and ESI commonly depends on the properties of the adsorbed layer.

From Table 4, PE showed the highest EAI (13.43 m^2^/g) compared to other proteins. Moreover, the lowest EAI was found in AE (2.48 m^2^/g). ESI ranged from 10.11 to 27.39 min, with AE having the highest ESI while SE and OSE displayed the lowest (10.11 and 10.48 min). The result implied that the extraction oil method had a noticeable impact on the EAI and ESI of FMS protein. The EAI values for FMS protein were better than the quinoa protein (4.2–7.4 m^2^/g) [36], but lower than that of pea protein (31.09–39.05 m^2^/g) [24]. Hydrophobic properties of proteins are the primary driving force for absorption at the oil-water interface and play a key role in the modification of emulsification properties. An increase in the protein hydrophobicity has been proved to improve the emulsifying properties [37]. The greatest EAI of PE may attributed to the potential unfolding of proteins in the pressing process, resulting in more small soluble protein exposed and adsorbed to the oil-water interface, thus providing better EAI. On the other hand, the Maillard reaction under high temperature caused partial denaturation of protein and changes in the charge and flexibility of the protein molecules, which could expose hydrophobic groups previously buried in their native conformation and improve the adsorption of the protein at the oil-water interface, thus leading to higher emulsifying properties [28]. Furthermore, the difference in the compositions and the amounts of the proteins as well as the aggregation of protein molecules also impact the emulsifying properties [38]. Wang et al. [39] reported that the reduction of emulsion stability of protein was related to the aggregation of protein molecules. SE and OSE showed lower ESI might be related to the higher levels of protein aggregates. Furthermore, the EAI is strongly related to the solubility, thus AE presented the lowest.

Furthermore, the change of amino acid composition and protein molecular structure also influenced the emulsifying properties. For example, the FMS protein extracted with organic solvents (petroleum ether and n-hexane) had similar EAI and ESI. This may be attributed to the similar amino acid composition (Table 2) and Mw of SE and OSE (Table 1).

## 4. Conclusions

The influences of oil extraction methods on FMS protein structure, amino acid composition, physicochemical properties and functional attributes were studied. FMS protein was rich in glutamic acid, leucine, aspartic acid, arginine, and proline, whereas cysteine was low. Extraction oil methods significantly impact the amino acid composition. SE exhibited the highest total amino acid contents, while AE had the minimum value. Oil extraction processes hardly influenced the Mw distributions of FMS protein but significantly impacted the protein aggregates contents. SE and OSE contained higher protein aggregates and small amounts of protein non-aggregates, whereas AE and PE showed opposite results. The peak denaturation temperatures of FMS protein ranged from 116.13 °C to 124.16 °C, indicating that it is a relatively thermally stable protein. Among these four FMS proteins, AE exhibited the highest water and oil holding capacity, PE had better solubility, foaming and emulsifying properties. The study provided useful insight into the impact of oil extraction methods on protein composition and structure, and highlighted the functional characteristics of FMS protein. Furthermore, a suitable oil extraction method should be chosen to produce both high-quality oil and protein, improving the utilization value of the FMS.

## Figures and Tables

**Table 1:** Yield, proximate composition and molecular weight distribution of field muskmelon seeds protein extracted by different oil extraction methods.

Sample	Yield (%)	Proximate Composition			Molecular Weight Distribution			
		Protein (%)	Moisture (%)	Fat (%)	Peak1 (kDa)	Peak2 (kDa)	C_aggregates_ (mg/mL)	C_non-aggregates_ (mg/mL)
SE	16.7	87.49 ± 0.45 ^a^	11.03 ± 0.19 ^a^	0.62 ± 0.03 ^c^	169.33 ± 3.48 ^a^	84.54 ± 1.44 ^ab^	8.01 ± 0.64 ^a^	1.99 ± 0.01 ^c^
OSE	16.3	73.12 ± 1.66 ^b^	6.02 ± 0.16 ^b^	10.26 ± 0.12 ^b^	172.89 ± 5.07 ^a^	79.95 ± 2.81 ^b^	7.29 ± 0.45 ^a^	2.71 ± 0.05 ^b^
AE	6.5	71.00 ± 0.15 ^b^	5.04 ± 0.12 ^b^	13.59 ± 0.08 ^a^	162.03 ± 1.22 ^a^	91.66 ± 1.37 ^a^	0.61 ± 0.03 ^b^	9.39 ± 0.18 ^a^
PE	3.3	73.62 ± 0.87 ^b^	6.09 ± 0.06 ^b^	9.28 ± 0.12 ^b^	168.92 ± 2.11 ^a^	87.31 ± 1.52 ^ab^	0.43 ± 0.01 ^b^	9.57 ± 0.13 ^a^

C_aggregates_, the amount of the protein aggregates; C_non-aggregates_, the amount of the protein non-aggregates. All values were expressed as mean ± standard deviation. Values with different superscripts within a column were significantly different (*p* < 0.05).

**Table 2:** Amino acid composition of field muskmelon seeds protein extracted by different oil extraction methods.

Amino Acid (mg/g)	SE	OSE	AE	PE
Cysteine	4.16 ± 0.03 ^a^	3.48 ± 0.14 ^b^	2.83 ± 0.08 ^c^	3.20 ± 0.04 ^b^
Valine	47.86 ± 4.30 ^a^	45.84 ± 2.28 ^a^	38.65 ± 2.84 ^a^	35.22 ± 0.64 ^a^
Methionine	43.08 ± 1.50 ^a^	38.69 ± 2.38 ^a^	39.11 ± 1.24 ^a^	43.84 ± 2.97 ^a^
Isoleucine	44.98 ± 1.44 ^a^	43.64 ± 0.51 ^a^	40.39 ± 1.97 ^ab^	36.88 ± 0.96 ^b^
Leucine	66.41 ± 0.85 ^a^	61.50 ± 2.29 ^ab^	56.23 ± 1.30 ^b^	52.88 ± 3.27 ^b^
Tyrosine	31.73 ± 2.45 ^a^	27.94 ± 1.41 ^ab^	24.43 ± 0.77 ^b^	23.96 ± 1.02 ^b^
Phenylalanine	55.06 ± 2.44 ^a^	47.27 ± 0.99 ^ab^	41.75 ± 2.31 ^bc^	38.60 ± 1.97 ^c^
Histidine	24.35 ± 1.56 ^a^	21.27 ± 1.79 ^ab^	18.40 ± 2.01 ^ab^	17.31 ± 0.71 ^b^
Lysine	26.09 ± 1.41 ^a^	22.56 ± 2.20 ^a^	20.55 ± 0.64 ^a^	21.02 ± 0.98 ^a^
Threonine	30.63 ± 1.63 ^a^	26.88 ± 1.59 ^ab^	23.57 ± 1.13 ^b^	21.72 ± 0.40 ^b^
Total essential amino acids	374.40 ± 4.61 ^a^	339.10 ± 6.90 ^ab^	305.95 ± 8.41 ^b^	293.18 ± 7.14 ^b^
Aspartic acid	73.77± 4.24 ^a^	65.96 ± 3.79 ^ab^	58.09 ± 2.66 ^b^	56.03 ± 2.09 ^b^
Serine	38.60 ± 1.56 ^a^	33.32 ± 1.26 ^ab^	29.76 ± 1.81 ^b^	28.47 ± 2.29 ^b^
Glutamic acid	125.61 ± 2.14 ^a^	111.17 ± 4.37 ^ab^	104.51 ± 4.32 ^b^	119.56 ± 4.33 ^ab^
Glycine	36.32 ± 3.05 ^a^	31.62 ± 2.87 ^a^	29.56 ± 1.43 ^a^	31.03 ± 1.12 ^a^
Alanine	38.68 ± 1.87 ^a^	32.03 ± 1.82 ^ab^	28.59 ± 2.25 ^b^	28.04 ± 2.78 ^b^
Arginine	59.39 ± 2.73 ^a^	52.65 ± 3.13 ^a^	50.04 ± 1.35 ^a^	57.76 ± 1.90 ^a^
Proline	56.57 ± 2.03 ^a^	45.86 ± 3.30 ^b^	44.16 ± 1.67 ^b^	42.82 ± 1.68 ^b^
Total non-essential amino acids	428.93 ± 4.50 ^a^	372.61 ± 4.60 ^b^	344.72 ± 4.65 ^c^	363.70 ± 5.20 ^bc^
Total amino acids	803.28 ± 7.07 ^a^	711.67 ± 7.24 ^b^	650.67 ± 5.55 ^c^	656.88 ± 5.66 ^c^

All values were expressed as mean ± standard deviation. Values with different superscripts within a row were significantly different (*p*< 0.05).

**Table 3:** Free sulfhydryl group contents and thermal properties of field muskmelon seeds protein extracted by different oil extraction methods.

Sample	SH (μmol/g)	T_o_ (°C)	T_d_ (°C)	T_e_ (°C)	ΔH (J/g)
SE	3.42 ± 0.01 ^d^	89.76 ± 4.10 ^a^	118.22 ± 3.05 ^a^	186.07 ± 0.78 ^a^	184.85 ± 4.03 ^a^
OSE	4.78 ± 0.03 ^c^	86.46 ± 5.28 ^a^	119.03 ± 4.87 ^a^	173.35 ± 4.43 ^b^	96.08 ± 4.92 ^b^
AE	8.86 ± 0.06 ^b^	92.28 ± 6.93 ^a^	124.16 ± 0.01 ^a^	176.29 ± 1.36 ^ab^	82.94 ± 0.35 ^c^
PE	12.60 ± 0.11 ^a^	84.36 ± 1.34 ^a^	116.13 ± 0.61 ^a^	176.29 ± 1.36 ^ab^	107.40 ± 0.28 ^b^

SH, free sulfhydryl group contents. T_o_, onset temperature; T_d_, denaturation temperature; T_e_, endset temperature; ΔH, enthalpy. ^c^ All values were expressed as mean ± standard deviation. Values with different superscripts within a column were significantly different (*p* < 0.05).

**Table 4:** Functional properties of field muskmelon seeds protein extracted by different oil extraction methods.

Sample	Solubility (%)	WHC (g/g)	OHC (g/g)	FC (%)	FS (%)	EAI (m^2^/g)	ESI (min)
SE	11.98 ± 0.06 ^c^	1.31 ± 0.01 ^b^	0.97 ± 0.01 ^c^	21.67 ± 2.89 ^a^	45.83 ± 7.22 ^b^	7.16 ± 0.09 ^b^	10.48 ± 0.05 ^c^
OSE	16.06 ± 0.08 ^b^	1.24 ± 0.02 ^b^	1.03 ± 0.03 ^bc^	12.50 ± 2.50 ^b^	32.78 ± 7.52 ^bc^	7.76 ± 0.09 ^b^	10.11 ± 0.02 ^c^
AE	10.65 ± 0.04 ^d^	1.48 ± 0.07 ^a^	1.36 ± 0.03 ^a^	12.50 ± 2.50 ^b^	17.22 ± 2.55 ^c^	2.48 ± 0.04 ^c^	27.39 ± 1.34 ^a^
PE	17.87 ± 0.08 ^a^	0.91 ± 0.01 ^c^	1.07 ± 0.02 ^b^	20.83 ± 3.82 ^a^	64.64 ± 6.01 ^a^	13.43 ± 0.08 ^a^	14.96 ± 0.14 ^b^

WHC, water holding capacity; OHC, oil holding capacity; FC, foaming capacity; FS, foam stability; EAI; emulsifying activity index; ESI, emulsion stability index. All values were expressed as mean ± standard deviation. Values with different superscripts within a column were significantly different (*p* < 0.05).

## Data Availability

Data is contained within the article.
